# E-waste for health: recycling smartphones for health monitoring

**DOI:** 10.3389/fdgth.2025.1495408

**Published:** 2025-06-11

**Authors:** Leandro Augusto de Almeida Costa, Brena Ataíde Furtado, Edilson Brabo Almeida, Bianca Callegari, Alex Harley Crisp, Rafael Oliveira Chaves, Maria da Conceição Nascimento Pinheiro, Givago Silva Souza

**Affiliations:** ^1^ Núcleo de Medicina Tropical, Universidade Federal do Pará, Belém, Brazil; ^2^ Instituto de Ciências da Saúde, Universidade Federal do Pará, Belém, Brazil; ^3^ Instituto de Tecnologia, Universidade Federal do Pará, Belém, Brazil

**Keywords:** digital health, portable digital devices, smartphones, electronic waste, health policy, health equity

## Abstract

Portable digital electronic devices have been widely used around the world for different everyday tasks. However, every day there is also an increasing dumping of these electronic devices, especially smartphones, creating a public health problem called electronic waste or e-waste. This Viewpoint discusses how e-waste could be used by governments to promote digital health policies, especially in poorer countries. The use of e-waste could lower health care costs and reduce exposure to the metals contained in these materials.

## Introduction

### Procedural historical background and development of e-waste

Since the early 1980s, the world, especially in highly urbanized areas, has experienced rapid development and commercialization of consumer electronic devices (1). This quickly led to a massive number of discarded devices, creating a public health concern now referred to as electronic waste, or simply e-waste. The issue of electronic waste (or e-waste) disposal has been a subject of concern since the early 2000s, with scholars identifying the growing challenges posed by those obsolete electronics when they come to end of cycle period or even end of life period (2).

This concern emerged prominently in large urbanized center around the world like Europe (3) and United States (1), due to increasing technological innovation and expanding consumer markets, particularly in relation to personal computers. These devices, characterized by their short life cycles, presented a growing environmental burden as landfilling and incineration became the dominant disposal methods. Until the mid-2000s, the primary focus in both Europe and the United States remained on personal computers.

Technological advancements have driven a shift from desktop computers to mobile devices and later to other portable devices (4), such as laptops and smartphones. With the accelerated release of new models, the phenomenon of pre-end-of-life obsolescence has intensified, wherein users replace devices for social, cultural, or aesthetic reasons rather than functional necessity. Xun Li and colleagues (2010) further demonstrates that repurposed smartphones can have a viable lifespan of at least five years in educational contexts, suggesting potential for extended use in other domains, such as healthcare applications (4). In different countries, the main reasons are not related to damage to the functioning of the device, indicating that the exchanged device would still be serviceable and, considering that even in devices exchanged for damage the damage is to a few components, a large part of the electronic components are still useful (5).

Electronic devices are now one of the most common items in homes around the world (6). Mobile phones, tablets and other portable electronic devices have taken on great importance in people's lives, especially in urban centers. Every year, new models are launched with aesthetic and technological novelties. Whether out of consumerism or necessity, exchanging devices for new models is a common behaviour, even before the end of the useful life of the current ones (7, 8). The average time smartphones are used is around 2 years (5), and many of them may still have some kind of functionality at the time of disposal (5). By 2022, according to the World Economic Forum, 5 billion mobile phones will have become e-waste (9).

More than 80% of all e-waste generated globally is still processed through informal management methods (10–12). Only a small proportion is treated using state-of-the-art formal recycling systems, which are designed to mitigate the most harmful effects of e-waste on human health and the environment (11, 12). When considering regional disparities, this imbalance becomes even more alarming, as most formal e-waste treatment facilities are concentrated in a limited number of typically well-developed countries (10). Additionally, there is a consistent flow of e-waste exported from developed to developing countries (10), exacerbating the already serious challenges related to improper e-waste disposal in regions that lack access to formal management systems. These regions often face compounded environmental and public health risks due to inadequate infrastructure and regulatory enforcement.

Germany, Japan, Canada, Australia, and the member states of the European Union exemplify national frameworks aligned with international Waste Electrical and Electronic Equipment (WEEE) conventions. Under these frameworks, producers, manufacturers, distributors, and importers are legally required to finance, organize, and operate systems for the collection, treatment, and recycling of WEEE. The regulations prescribe specific collection targets and recycling rates, mandate producer registration and annual performance reporting, and incentivize ecodesign and the development of products that facilitate both disassembly and material recovery (11).

The situation in many developing countries remains significantly worse and far from the standards observed in developed nations. Countries such as Ghana, Nigeria, Thailand, India, Brazil, and Vietnam continue to receive substantial volumes of e-waste exported from other regions, exacerbating their existing treatment burdens (12, 13). Although global guidelines and international and regional policies seek to curb these transboundary movements, compliance often falls short, since not all jurisdictions include strict export-restriction measures in their national e-waste regulations (11, 12).

Consequently, the proportion of e-waste managed through informal channels in these settings is typically higher than in developed contexts. This trend stems both from insufficient resources for formal collection and recycling infrastructure and from the lucrative incentives associated with recovering valuable subcomponents through unregulated management practices. Such informal handling magnifies the adverse impacts on human health and the environment in these regions (12).

Within this landscape, reuse emerges as a key strategy consonant with circular-economy principles for managing electronics at their end of life when obsolescence arises from factors other than complete or partial functional failure [see the categories of mobile-phone obsolescence in Wilson et al., 2017 (14)]. Devices that are no longer required are frequently relegated to storage or hibernation rather than formally recycled (9). By extending the in-use lifespan of these products through well-structured reuse programs, stakeholders can substantially mitigate improper disposal practices (15). Moreover, reuse initiatives afford critical time for the development and implementation of formal recovery and recycling systems, ensuring that when final disposal becomes unavoidable, it occurs under conditions that minimize environmental and health risks.

### How modern does a smartphone have to be to serve as a support tool for monitoring people's health?

Smartphones in general are validated for a series of measurements of biological signals that can be used in health care practices (16). They are equipped with a set of sensors and components such as accelerometers, gyroscopes, touchscreens, cameras and LEDs. All these components make it possible to make specific assessments that can be useful in the case of diagnoses or the assessment of health conditions if correctly used in conjunction with healthcare professionals. What's more, these components are generally not among the components that are unserviceable when the device is disposed of (17), opening up a range of possibilities for their reuse in healthcare practices.

Older smartphones often become obsolete for use by users in the face of cultural and market developments. Modifications to the memory and processing of smartphones change from one generation to the next as current applications demand more and more storage capacity and information processing. However, the rest of the electronic components remain relatively close from one generation to the next, opening up the possibility that they can be reused for the purposes outlined in this article without significant damage.

One of these smartphones discarded for trivial use, which can be formatted, has the potential to be a tool for collecting diagnostic and evaluative data on health conditions. Some precautions are probably necessary, such as ensuring that it is used exclusively for the task related to data collection, avoiding adding extra functions to the device, such as making it for personal or recreational use by the health professional using it or other people with access to it. It is recommended that it be used exclusively for this purpose, preventing the installation of other applications unrelated to the task for which it is to be used. As these are devices designed for general purposes, and not specifically for the function suggested here, such precautions are necessary to minimise the chance of unexpected behaviour of the device's physical or digital components leading to variations in the quality of the data collected.

### Practical aspects and examples of policies and practical initiatives concerning e-waste for health

The main reference for policies related to the implementation of mobile digital tools in healthcare has been led by the World Health Organization (WHO). The WHO proposed a Global Digital Health Strategy for 2020–2025 (18), aiming to strengthen health systems through the application of digital health technologies for consumers, health professionals, healthcare providers, and the industry, ultimately empowering patients and advancing the vision of “health for all.” The strategy includes recommendations based on a critical evaluation of the evidence surrounding emerging digital health interventions, such as: birth and death notifications via mobile devices, stock notification and commodity management via mobile tools, telemedicine, clinical decision support for health workers via mobile devices, digital tracking of patients'/clients' health status and service use, and remote training for health workers using mobile technologies (19). These interventions have contributed to improvements in health systems and were assessed in terms of benefits, risks, acceptability, feasibility, resource use, and equity considerations.

Recently, Kulkarni and colleagues (2022) conducted a review of empirical studies on e-health practices utilizing smartphones. A total of 71 studies were collected based on the defined criteria for the review, covering the period from 2017–2022 (20). Among these, 4% were classified by the authors as related to chronic diseases (e.g., Parkinson's disease), 37% to the wellness domain (e.g., sleep, physical activity, sociability, drug use, and diet), and 59% to the mental health category (e.g., general mental health, stress, schizophrenia, mood disorders, depression, and bipolar disorder). The review also highlights the sensors and smartphone functionalities employed, along with their potential advantages and disadvantages. Key points include: (1) accelerometers and gyroscopes as low-power, highly private and high sensitive sensors, particularly useful for detecting both the quantity and quality of general physical activity; (2) cameras and microphones as tools for capturing direct environmental data such as sound and lighting, in addition to inferred information regarding sociability, mobility, and other behavioral patterns; (3) Global Positioning System (GPS) for direct location monitoring and other indirect inferences; and (4) text messaging and call log data as direct measures of the quantity and quality of remote interactions, which are also valuable for assessing well-being and monitoring social and mental health states (20).

In monitoring high-risk pregnancies, smartphones play a role in maternal health management. Mobile applications can provide expectant mothers with guidance, reminders for prenatal care, and communication with healthcare providers. Wearable sensors connected to smartphones help track vital signs such as blood pressure and fetal heart rate, enabling the detection of complications and medical interventions. These technologies are particularly relevant in low-resource settings, where access to specialized obstetric care is limited (21).

For mental health, mobile phone-based interventions have demonstrated potential in supporting patients with psychiatric disorders. Digital tools offer remote therapy sessions, cognitive behavioral therapy modules, and mental health tracking features, allowing individuals to manage and share their conditions. Mobile-based interventions have been beneficial in reducing psychological distress and improving quality of life. However, challenges such as heterogeneity in intervention efficacy and publication bias indicate the need for evaluation frameworks to assess their impact (22).

As highlighted by Domin and colleagues (2021), mobile applications designed for physical activity (PA) interventions contribute to behavior change by incorporating features such as step tracking, goal setting, and motivational feedback. While a number of studies support the efficacy of smartphone-based PA interventions, the absence of standardized evaluation frameworks hinders assessments of their impact. The authors also emphasize that the development of systematic methods for designing and evaluating PA applications is necessary for improving their effectiveness (23).

For patients with Parkinson's disease, smartphone-based technologies offer solutions for symptom tracking and intervention (24, 25). Mobile applications can analyze movement patterns, tremors, and gait abnormalities using built-in sensors, allowing for remote monitoring and detection of disease progression. Digital platforms accessible on these devices can also provide cognitive and motor exercises tailored to individual needs, supporting patient adherence to therapeutic regimens. These tools facilitate communication between patients and healthcare providers, enabling adjustments to treatment plans and improving disease management.

In conclusion, smartphones have influenced healthcare by expanding access to medical services, contributing to chronic disease management, and supporting patient engagement. Their role in high-risk pregnancy care, mental health interventions, physical activity promotion, and Parkinson's disease management highlights their versatility and application. However, some of the cited reviews also highlight the need to improve the robustness of the studies presented, particularly regarding methodological rigor in demonstrating the effectiveness of certain interventions (22). Additionally, they emphasize the necessity of a conducive environment for the implementation of e-Health practices, including technological fluency among both healthcare professionals and patients, as well as adequate funding for the material implementation of these initiatives (26), which, in some contexts, requires governmental support for funding and dissemination of the measures to be implemented.

### Potential contribution to the equity of public health measures in underdeveloped and developing countries

In addition to the contribution of reusing obsolete devices to sustainable demands for e-waste disposal, we also see the practice as a possible contributor to the equity of public health measures in countries that have historically faced difficulties in providing large-scale care in some countries, especially in their localities that are further away from urban centres or have less financial investment. As some diagnostic and evaluative methods in health can be costly and difficult to implement in these contexts, having alternative and validated large-scale implementation options for smartphones can help contribute to a greater range of care for the population in these areas. This is because acquiring a smartphone in usable condition for certain types of assessment, especially if it comes from recycling or waste, tends to be much cheaper than obtaining equipment developed and marketed specifically for the health area.

Various studies around the world have proposed applications aimed at providing health information for the public health system, for use in different hospital tasks, rehabilitation assistance, monitoring physical activity levels, telemedicine and mobility assessment in the home environment, among others (27–32). The arrival of smartphones to provide functionalities in these various environments of the health care system could make up for the lack of instruments considered gold standards for these functionalities which, due to their high financial cost, should not even be considered for acquisition of the service, especially in primary health care.

In addition to all the advantages for people's health care, the reuse of obsolete smartphones would make it possible to reduce the number of these devices in rubbish dumps, which can release heavy metals into the environment and lead to the exposure of people to these metals, which can lead to a variety of toxicities (33).

### How governments, especially in poor or developing countries, could act to reuse e-waste that is still usable in favour of programmes aimed at health care practices

Health administrators in poor and developing countries could take action to set up programmes to collect these discarded or discarded smartphones that make up this mountain of electronic waste. The devices could be assessed by technicians to check their viability and thus be reused, potentially for more than one purpose such as education and health, or even distributed to the population that doesn't have access to these technologies. It would depend on the technical assessment. And those that are suitable for use in the health sector would be properly allocated to the appropriate areas and professionals.

### Novelty of the proposal

The reuse of smartphones for health assessment introduces an approach that integrates environmental sustainability based on CE with digital health innovation. While smartphones are already widely used in health-related applications, the core of this proposal lies in repurposing devices that would otherwise be discarded as electronic waste (e-waste) and using them as low-cost, scalable tools for diagnostic and evaluative purposes in healthcare.

Unlike traditional digital health initiatives that depend on modern and often expensive devices, this approach focuses on older smartphones that have become obsolete in consumer markets, yet still retain fully functional sensors and hardware components (such as accelerometers, gyroscopes, cameras, and microphones) that are essential for health monitoring. These components are typically not the ones that fail when the device is discarded, making them suitable for tasks such as motion analysis, physical activity tracking, symptom monitoring (e.g., tremor or gait in Parkinson's disease), and even telehealth communication.

This reuse model bridges the digital health gap in underserved areas by offering affordable tools for health services; reduces e-waste by expanding the usable time of devices, contributing to environmental sustainability and addressing public health risks associated with improper disposal; proposes the exclusive reconfiguration of smartphones for clinical use (e.g., restricting apps and functionalities), minimizing variability in data collection due to unrelated usage; and suggests a systematic pathway for governments and health systems to integrate e-waste recovery into public health strategies, particularly in resource-limited settings.

Moreover, this initiative aligns with the principles of the circular economy (34), which emphasizes the extension of product lifecycles, resource efficiency, and the reintegration of discarded materials into productive use. By repurposing smartphones for healthcare, the model shifts from a linear “take-make-dispose” logic to a circular system where electronic devices continue to generate value beyond their initial consumer lifespan, contributing simultaneously to technological equity and environmental stewardship.

The proposal also has limitations, particularly concerning the financial and logistical costs associated with collecting and configuring smartphones for reuse in clinical or home settings. These costs are not addressed in the present study but would need to be carefully considered by healthcare managers prior to implementation. Additional concerns include how the biological data collected via smartphones would be transferred to healthcare professionals for evaluation, as well as where the large volumes of data would be securely stored for future analysis. Ethical issues related to data privacy and the protection of personal identity must also be taken into account. A more feasible initial strategy may involve implementing the intervention in small communities, accompanied by ongoing evaluation of its outcomes, before considering broader expansion to larger healthcare centers.

## Conclusion

In summary, this proposal is not merely in using smartphones for health monitoring, which is well documented, but in strategically repurposing obsolete devices to extend their functional life and expand access to digital health tools, especially where high-cost solutions are not feasible. It's a synergistic approach that tackles both technological waste and inequities in healthcare access.

Recycling smartphones for healthcare is an action that can bring direct and indirect benefits to the population. Countries can pilot this type of digital intervention to identify difficulties and propose improvements. However, action needs to be taken because the potential for gains in people's health and savings in spending on people's health and private and public healthcare services can be quite significant.

We do not believe that repurposing smartphones for health assessments will fully resolve the issue of e-waste. However, we consider this strategy to be a potentially valuable component of a broader governmental action plan, developed in collaboration with device manufacturers and society, that aims to reduce waste by extending the useful life of electronic devices. Such a plan would also involve public education on proper use and disposal practices, as well as the appropriate management of discarded devices, including their potential application in the healthcare sector.

## Data Availability

The original contributions presented in the study are included in the article/Supplementary Material, further inquiries can be directed to the corresponding author.
